# Influence of the DRD2/ANKK1 Taq1A polymorphism on caudate volume in older adults without dementia

**DOI:** 10.1007/s00429-018-1650-0

**Published:** 2018-03-21

**Authors:** Xin Li, Goran Papenberg, Grégoria Kalpouzos, Lars Bäckman, Jonas Persson

**Affiliations:** Aging Research Center (ARC), Karolinska Institute and Stockholm University, Gävlegatan 16, 113 30 Stockholm, Sweden

**Keywords:** Dopamine, D2 receptors, Caudate, Aging, ANKK1, Taq1A

## Abstract

Dopaminergic neuromodulation is critically important for brain and cognitive integrity. The DRD2/ANKK1 Taq1A polymorphism is associated with striatal dopamine (DA) D2 receptor availability. Some previous studies have found that the A allele of the Taq1A polymorphism influences brain structure, but the results are inconsistent, likely due to population heterogeneity and small sample sizes. We investigated the genetic effect on caudate volume in a large sample of older adults without dementia. Results show that A-allele carriers have smaller caudate volume compared to non-carriers in relatively older adults (*n* = 167; Mage = 77.8 years), whereas the genotype did not influence caudate volume in a younger age group (*n* = 220; Mage = 62.8 years). Cognitive performance was not significantly affected by the DRD2 gene. Our findings extend previous observations by showing magnified genetic effects on brain volume in old age, and provide evidence for a link between a DA-related genetic polymorphism and grey matter volume in a brain region within the nigrostriatal dopaminergic pathway.

## Introduction

The neurotransmitter dopamine (DA) plays a critical role in human cognition. The DA D2 receptor has the highest density in the striatum, including the caudate and putamen, and is sparsely distributed in the neocortical and limbic regions (Camps et al. 1989; Hall et al. 1994). Caudate DA levels are linked to higher cognitive processes, such as episodic memory, working memory, habitual learning and cognitive switching (Cervenka et al. 2008; Cools 2011; Bäckman et al. 2011; Nyberg et al. 2016). Structural and functional MRI studies show that DA D2 receptor levels are positively related to caudate volume (Woodward et al. 2009) and may contribute to cognitive performance by influencing functional connectivity between caudate and the hippocampus (Nyberg et al. 2016).

The DA D2 receptor is encoded by the DRD2 gene, which is closely linked to the ANKK1 gene (Neville et al. 2004). The Taq1A polymorphism (rs1800497) is located about 10 kb downstream from the DRD2 gene and lies within the ANKK1 gene. Individuals who carry the A allele have reduced D2 receptor density in the caudate and putamen compared to non-carriers (Pohjalainen et al. 1998; Jönsson et al. 1999; Thompson et al. 1997). The presence of the A allele has been related to worse performance on executive function tasks (Persson et al. 2015; Berryhill et al. 2013; Jocham et al. 2009). However, as some studies have found no effect (Cerasa et al. 2009) or even a reversal effect (Bartres-Faz et al. 2002; Tsai et al. 2002) of the Taq1A polymorphism on cognition, the association between the DRD2 gene and behavioral performance still remains unclear.

Aging is associated with changes in dopaminergic neurotransmission (for a review, see Reeves et al. 2002). Compared with younger adults, older adults show neuronal losses (Fearnley and Lees 1991), decrease in biosynthetic enzymatic activity (Martin et al. 1989), lower DA transporter availability (Volkow et al. 1998b; Erixon-Lindroth et al. 2005), reduction of striatal D1 (Rieckmann et al. 2011; Wang et al. 1998) and D2 (Bäckman et al. 2000; Volkow et al. 1998a) receptor binding, as well as decline of endogenous DA (Kish et al. 1992). A recent meta-analysis of cross-sectional PET and SPECT studies found reliable negative effects of age on D1 and D2 receptors and DA transporters, but a spared DA synthesis capacity with age, which might act as a compensatory mechanism for reduced DA receptor binding (Karrer et al. 2017). An increasing number of studies have found that the effects of genetic variations on cognition, brain structure, and brain function may be magnified in aging (for a review, see Papenberg et al. 2015a). The resource-modulation hypothesis assumes that genetic influences on cognition are more pronounced for individuals with limited neuroanatomical or neurochemical resources, such as older adults (Lindenberger et al. 2008). This hypothesis is based on the assumption that the function relating brain resources to cognitive performance is non-linear (Cools and D’Esposito 2011; Li and Sikstrom 2002). According to this hypothesis, with a steeper slope of the brain resources loss during normal aging, the effect of genetic variation should increase from young to younger-old and to older-old age. As the brain resources continue to decrease and are depleted in individuals with dementia and terminal decline, genetic effects are expected to diminish again.

Very few studies have investigated the influence of the Taq1A polymorphism on behaviour and brain in older adults. However, extant research supports the resource-modulation hypothesis, demonstrating magnified genetic effects in older compared to younger adults (Papenberg et al. 2013, 2014, 2015b; Colzato et al. 2013). Also, Persson et al. (2015) found that, compared to non-carriers, older A-carriers had worse memory updating performance and exhibited less blood oxygen level-dependent (BOLD) activation in the left caudate nucleus, a region critical for updating (Dahlin et al. 2008; Bäckman et al. 2011; O’Reilly and Frank 2006). These effects were not seen in younger adults.

Conceivably, the cell numbers reflected by a volumetric measure in a brain structure are important for capillary recruitment associated with functional activity (Makris et al. 2004). Thus, there may be an association between reduced brain activity and reduced volume in the same area, and it is therefore expected that A-carriers have smaller caudate volume than non-carriers. However, few studies have investigated the effect of the DRD2 A allele on caudate volume, and the results are inconsistent. For example, Bartres-Faz et al. (2002) found that A-carriers had larger caudate volume compared to G-carriers in 49 memory-impaired participants. Other studies failed to find any A-allelic effect on the caudate in 70 younger adults (Cerasa et al. 2009), and in persons with mild cognitive impairment and dementia (Stein et al. 2011). This inconsistency may reflect the relatively small sample sizes and the heterogeneity of the investigated samples. As predicted by the resource-modulation hypothesis (Lindenberger et al. 2008; Papenberg et al. 2015a), the association between genes and different phenotypes may be weaker in younger adults and individuals with dementia compared with normal older adults. To address these possibilities, we explored the interaction between the DRD2/ANKK1 Taq1A polymorphism and age on caudate volume in a large sample of older adults without dementia (*n* = 387). Because the potential genetic effect may be stronger in relatively older adults, we stratified the sample by age. As the presence of an A allele of the DRD2/ANKK1-TaqIA polymorphism is related to reduced density of DA D2 receptor densities (Pohjalainen et al. 1998; Jönsson et al. 1999; Thompson et al. 1997), and DA D2 receptor levels are positively related to caudate volume (Woodward et al. 2009), we hypothesized that A-carriers may have smaller caudate volumes compared with non-carriers. In accordance with the resource-modulation hypothesis, we further expected that this effect is larger in older compared to younger age groups. We also investigated the impact of gene, age, and their interaction on cognitive performance.

## Methods

### Participants

The sample consisted of 556 participants (age range = 60–96 years; mean age = 70.9 years) from a population-based study, the Swedish National Study on Aging and Care in Kungsholmen (SNAC-K). Parts of the data from SNAC-K have been published elsewhere, along with more detailed descriptions of the project (Lövdén et al. 2013; Laukka et al. 2013; Papenberg et al. 2015b). The initial SNAC-K sample (*n* = 3363) was stratified by age (60, 66, 72, 78, 81, 84, 87, 90, 93, 96, and 99 years) at baseline. A subsample of noninstitutionalized and nondisabled participants was randomly selected to further undergo structural magnetic resonance imaging (MRI). Participants with Parkinson’s disease, self-reported stroke, stroke observed on the MR images, epilepsy, schizophrenia, and bipolar disorder were excluded. Dementia was diagnosed using a three-step procedure to diagnose according to the Diagnostic and Statistical Manual of Mental Disorders, 4th edition (DSM–IV; American Psychiatric Association, 1994). First, an examining physician made a preliminary diagnosis, and this diagnosis was compared with a second independent diagnosis, which was based on computerized data only. In cases of disagreement, a supervising physician made a third and final diagnosis. The cognitive assessment used in the diagnoses included the MMSE (Folstein et al. 1975), the Clock test (Manos and Wu 1994) and items regarding memory, orientation, executive functioning, interpretation of proverbs, and problem solving. The physicians were blind to the results of the cognitive test battery, which was administered at a separate session. We excluded participants with a dementia diagnosis at baseline or at either of the two follow-ups (after 3 and 6 years). 27 participants were omitted due to missing genetic data. Eight participants, 90 years and older, who likely represent a positively selected subgroup relative to the full study sample were excluded to have more homogenous age groups in the analyses. The effective sample for the current study consisted of 387 participants (age range = 60–87 years; mean age = 69.3 years). The number of participants in each age group, along with information on the number of A-carriers in each age group, is shown in Table 1. As the number of subjects older than 72 years is relatively small, we combined them together and stratified the whole sample into two age groups, 60–66 years (younger old) and 72–87 years (older old) for each age group to contain an equal number of participants.

**Table 1:** Number of subjects across different age and gene groups

	Age							Total
	60	66	72	78	81	84	87	
DRD2/ANKK1								
A−	88	63	37	29	23	12	9	261
A+	31	38	19	22	6	8	2	126
Total	119	101	56	51	29	20	11	387

Demographic and genetic characteristics across age and genotype groups are shown in Table 2. There were no significant differences in age, sex, or education between A allele carriers and non-carriers in either the total sample or within the two age groups. The mean ages of the younger-old and older-old age groups were 62.8 and 77.8 years, respectively. Educational level and MMSE scores were higher in the younger-old age group. There was no sex ratio difference between the two age groups. Besides, no difference in the allelic distribution for the APOE or COMT genes was found between age or ANKK1 groups.

**Table 2:** Demographic and genetic information across age and DRD2/ANKK1 genotype groups

	Younger-old group				Older-old group				All (*p*-values)	
	All	A+	A−	*p*	All	A+	A−	*p*	Y versus O old	A+ versus A−
*N*	220	69	151		167	57	110			
Age, years	62.8	63.3	62.5	0.07	77.8	77.5	78.0	0.51	< 0.001	0.46
Gender (f/m)	128/92	45/24	83/68	0.15	103/64	34/23	69/41	0.70	0.49	0.40
Education, years (range)	13.9 (7–28)	14.2	13.8	0.53	11.6 (5–25)	11.3	11.8	0.46	< 0.001	0.87
MMSE (range)	29.4 (25–30)	29.4	29.4	0.84	29.0 (25–30)	29.0	29.0	0.87	< 0.001	0.89
APOE any ε4	26%	27.5%	25.3%	0.73	22.4%	25%	21.1%	0.57	0.42	0.54
COMT any val	71.2%	63.2%	74.8%	0.08	69.8%	75.5%	67%	0.27	0.77	0.57

### Cognitive measures

All participants underwent a battery of cognitive tests covering five domains that included: perceptual speed—digit cancellation and pattern comparison; episodic memory—verbal free recall and cognition; semantic memory—vocabulary and general knowledge; and verbal fluency—letter fluency and category fluency. Below, we briefly describe the cognitive tasks, and more details can be found in Laukka et al. (2013).

#### Perceptual speed

Perceptual speed was assessed using two paper-and-pencil tests: digit cancellation (Zazzo 1974) and pattern comparison (Salthouse and Babcock 1991). Digit cancellation consisted of 11 rows of random digits ranging from 1 to 9. Participants were instructed to sequentially go through the rows as quickly as possible and cross out every “4” they encountered. For pattern comparison (Salthouse and Babcock 1991), participants were presented with pairs of line segment patterns. They were asked to sequentially go through the patterns as rapidly as possible and mark whether the patterns were the “same” or “different.”

#### Episodic memory

Episodic memory testing involved free recall and recognition, and was assessed using a word list comprising 16 unrelated concrete nouns, presented both orally and visually. Participants were instructed to memorize the words. Immediately after presentation, they were given 2 min for oral free recall. Following free recall, participants were given a self-paced recognition task, where the 16 target words were presented randomly intermixed with 16 lures. Participants were first asked whether they recognized the word from the first presentation and then to distinguish whether they could recollect the word or if the word was merely familiar. Recognition scores were based solely on recollection responses.

#### Semantic memory

Two semantic memory tasks that measure word comprehension and general knowledge, respectively, were administered. The SRB:1 is a revised 30-item vocabulary test (Dureman 1960; Nilsson et al. 1997), where each target word is presented together with five other words. Participants were instructed to underline the word representing the synonym to each target word. The general knowledge task consisted of ten questions (e.g. “What is the capital of Uruguay?”) that have been found to be moderately difficult for older people (Dahl et al. 2009). Participants were instructed to select the correct answer out of two alternatives.

#### Letter fluency

Participants were asked to orally generate as many words as possible, beginning with the letters F and A, respectively. They were given 60 s for each task and they were instructed that proper names, numbers, or words with a different suffix were not credited.

#### Category fluency

Participants were asked to orally generate as many words as possible within 60 s, belonging to the categories of animals and professions, respectively.

### Genotyping

DNA was extracted from peripheral blood samples using standard methods. The DRD2 rs1800497 SNP (Taq1A) was genotyped using MALDI-TOF analysis on the Sequenom MassARRAY platform at the Mutation Analysis Facility, Karolinska Institutet (Darki et al. 2012). In the present sample, 67.4% participants with GG genotype were categorized as A− allelic status group; 28.4% participants carrying the AG genotype and 4.1% carrying the AA genotype were combined and categorized as A+ allelic status group. The rs1800497 distribution was in Hardy–Weinberg equilibrium (*p* > 0.01). As previous research has found that the catechol-*O*-methyltransferase (COMT) Val158Met polymorphism (rs4680) and apolipoprotein E (APOE) (defined by two SNPs, rs429358 and rs7412) polymorphisms can influence caudate volume (Liu et al. 2010b; Watanabe et al. 2015), we also genotyped these two polymorphisms to identify any potential confound in interpreting DRD2 effects on caudate volume.

### MRI acquisition

All MRI measurements were conducted using a whole-body clinical 1.5 T MRI scanner (Philips Intera, The Netherlands). 3D FFE (fast field echo) T1, axial SE (spin echo) PD/T2, axial FLAIR (fluid-attenuated inversion recovery), and axial diffusion tensor imaging were acquired. In this study, the axial 3D FFE T1 images (TR = 15 ms, TE = 7 ms, flip angle = 5°, number of slices = 128, slice thickness = 1.5 mm, in-plane resolution = 0.94 × 0.94 mm^2^, no gap, FOV = 240, Matrix = 256 * 256) were used to derive volumetric data.

### VBM analyses

T1-weighted MR images were segmented into grey matter, white matter, and cerebrospinal fluid using the unified segmentation approach (Ashburner and Friston 2005) in SPM12b (Statistical Parametric Mapping, Wellcome Trust Centre for Neuroimaging, http://www.fil.ion.ucl.ac.uk/spm/) implemented in Matlab 10 (The Mathworks, Inc). The “light cleanup” option was used to remove odd voxels from the segments. The grey matter images were further analyzed using DARTEL in SPM12b (Diffeomorphic Anatomical Registration Through Exponentiated Lie Algebra, Ashburner 2007). The grey matter segments were imported into DARTEL space, and a customized grey matter template was created including subject-specific flow fields containing the individual spatial normalization parameters (diffeomorphic non-linear image registration). By incorporating the affine transformation of the DARTEL template to Montreal Neurological Institute (MNI) space, the grey matter segments were further warped into standard MNI space. To preserve local-tissue volumes, the normalized grey matter volumes were modulated by scaling them with Jacobian determinants from the registration step. Volumes were smoothed with a full-width at half maximum Gaussian kernel of 9 mm in three directions.

We defined two masks of left and right caudate as regions of interest, which were used to extract normalized volumes from each individual’s smoothed images. The masks were based on the WFU Pickatlas AAL. We then visually inspected the fit of each mask to the mean grey matter mask and optimized each mask’s fit in terms of clear separations between masks. The resulting masks were then used to extract mean regional volumes for each participant. To test for regional and lateral specificity of age and gene effects on caudate volume, we also created masks and extracted brain volumes from both hemispheres for seven additional brain regions (Table 3). Intracranial volume (ICV) was used to adjust volumetric data based on the analysis of covariance approach: adjusted volume = raw volume − *b* (ICV−mean ICV), where *b* is the slope of regression of volume on ICV (Jack et al. 1989; Raz et al. 2005). We aggregated the adjusted volumetric data of the left and right caudate as caudate volume. All variables displayed acceptable skewness and kurtosis (Kline 2005).

**Table 3:** Effects of genotype and age (*p* values) on brain regions

	Cd	Calc	Hc	IPL	IT	LPFC	OFC	Pt
Age	< 0.01	< 0.01	< 0.01	< 0.01	< 0.01	< 0.01	< 0.01	0.409
DRD2	0.163	0.539	0.246	0.535	0.791	0.687	0.328	0.633
Age × DRD2	0.09	0.529	0.584	0.985	0.484	0.927	0.49	0.116
Laterality × age × DRD2	0.022	0.874	0.55	0.876	0.922	0.134	0.236	0.989

*Cd* caudate nucleus, *Calc* calcarine sulcus, *Hc* hippocampus, *IPL* inferior parietal lobule, *IT* inferior temporal cortex, *LPFC* lateral prefrontal cortex, *OFC* orbito-frontal cortex, *Pt* putamen

### Statistical analyses

All data were analyzed using IBM SPSS 21.0. Analyses of covariance (ANCOVA) were conducted to investigate the effects of age (60–66 years versus 72–87 years), DRD2/ANKK1 Taq1A allele load (GG versus any A), and their interaction on caudate volumes and cognitive performance. We further performed an analysis that included both hemispheres of caudate volume to examine the effects of laterality using a repeated-measures ANCOVA (left, right) × age (younger, older) × gene (A carriers, non-carriers). ANCOVAs were also performed on seven additional brain regions as a control analysis to test the specificity of the age by gene interaction on caudate volume. Performance in the five cognitive domains was calculated as the mean scores of two cognitive tasks within each domain. As ANKK1 and age groups did not differ on sex distribution and allelic distribution of APOE and COMT gene, only education was used as a covariate in the statistical analyses. Post hoc pairwise comparisons were performed for significant interactions. A *p* value of < 0.05 was considered significant. Effect sizes are indicated by partial eta squared (*η*^2^_p_).

## Results

### Genetic effects on grey matter volumes

**Fig. 1:**
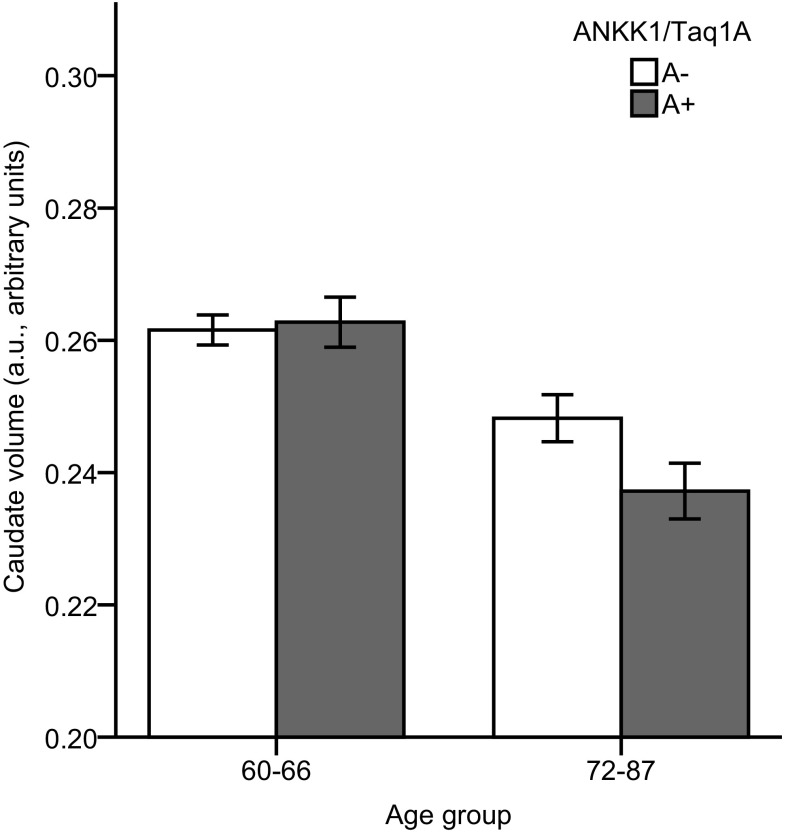
Mean adjusted caudate volume for the younger-old and older-old age groups, and ANKK1/Taq1a A-carriers (dark grey bars) and non-carriers (white bars). Error bars show standard error of the mean

**Fig. 2:**
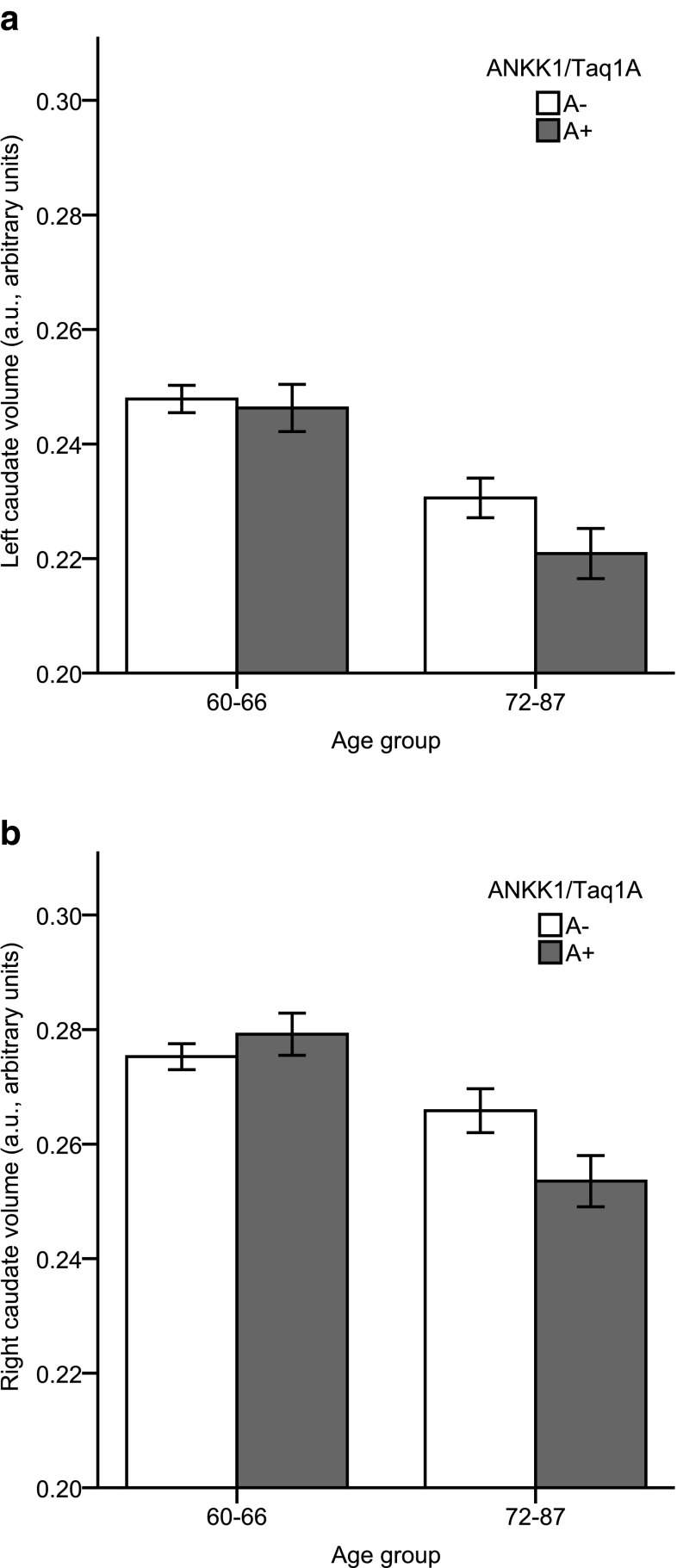
Mean adjusted left (**a**) and right (**b**) caudate volume for the younger-old and older-old age groups, and ANKK1/Taq1a A-carriers (dark grey bars) and non-carriers (white bars). Error bars show standard error of the mean

We found a significant main effect of age in the caudate nucleus (*p* < 0.001), indicating smaller volumes in the older age group. Moreover, there was a trend for a gene × age interaction in the caudate (*F* (1,386) = 2.89, *p* = .09, *η*^2^_p_ = 0.008) reflecting smaller caudate volume in A-allele carriers compared to non-carriers in the older-old group only (Fig. 1). The repeated-measured ANCOVA revealed a significant main effect of age (*p* < 0.001) and a significant laterality × age × gene interaction (*p* = 0.022) on caudate volume. Critically, there was a reliable gene × age interaction in the right caudate nucleus *F* (1,386) = 4.86, *p* = 0.028, *η*^2^_p_ = 0.013, but not in the left caudate nucleus showing that the interaction on caudate was mainly driven by the right caudate. Pairwise comparison revealed that there was a significant gene effect for the right caudate region in the older age group (*p* = 0.025), but not in the younger age group (*p* = 0.428), reflecting that A-allele carriers had a significantly smaller right caudate volume compared with non-carriers in the older-old group only (Fig. 2).

We assessed the specificity of the effects on caudate volume by conducting separate ANCOVAs on seven cortical and subcortical structures. As shown in Table 3, the laterality × age × gene interactions are significant (marginally significant for age × gene interaction) in the caudate, but fall short of significance for all other regions tested. These findings suggest that the genetic effect is restricted to the caudate.

### Genetic effects on cognition

As shown in Table 4, there were significant main effects of age for three cognitive domains: perceptual speed, episodic memory, and category fluency (all *p*s < 0.005). There was a trend showing that A-carriers performed somewhat better on episodic memory, but neither the main effect of ANKK1 status nor the interaction between age and ANKK1 was statistically significant for any cognitive measure.

**Table 4:** Effects of genotype and age (*p* values) on cognitive performance

	DRD2	Age	Interaction
Perceptual speed	0.71	< 0.001	0.30
Episodic memory	0.06	0.001	0.53
Semantic memory	0.83	0.84	0.18
Letter fluency	0.76	0.96	0.29
Category fluency	0.62	< 0.001	0.27

## Discussion

We examined the effect of the Taq1A polymorphism of the DRD2/ANKK1 gene on caudate structure and cognitive performance in older adults without dementia. There was an age-related deficit in perceptual speed, episodic memory, and verbal fluency, although the younger-old and older-old groups did not differ in semantic memory or letter fluency. These patterns are consistent with prior research (for reviews, see Bäckman et al. 2001; Hoyer and Verhaeghen 2006). There was no significant influence of the DRD2 gene on cognitive performance. Older age was associated with smaller caudate volume, which confirms previous reports (Raz et al. 2003, 2005; Taki et al. 2013; Papenberg et al. 2016; Gunning-Dixon et al. 1998). Most importantly, in the older subgroup, A-carriers had smaller caudate volume compared to non-carriers, whereas there was no such effect in the younger subgroup. The magnified genetic effect on caudate volume in older-old persons is consistent with the resource-modulation hypothesis, which posits that genetic effect may be larger when brain resources are reduced, such as in aging. Our results extend previous findings showing larger genetic effects in older adults on cognition (Papenberg et al. 2014; Liu et al. 2010a; Colzato et al. 2013; Persson et al. 2015), brain activity (Persson et al. 2015), and white matter integrity (Papenberg et al. 2015b) to also include effects on grey matter morphology.

The underlying cause of the genetic effect on caudate volume remains to be specified. The association between the ANKK1 polymorphism and caudate volume may be accounted for by the role of this polymorphism in striatal receptor density (Bartres-Faz et al. 2002; Cerasa et al. 2009; Hirvonen et al. 2004). The caudate nucleus is an important region in the nigrostriatal pathway, in which DA is transmitted from the substantia nigra to the striatal complex. The ANKK1 Taq1A allele has been related to lower DA D2 receptor density in caudate and putamen (Pohjalainen et al. 1998; Jönsson et al. 1999), and may thus be linked to reduced DA synthesis in the dorsal striatum. As DA has trophic effects (Nieoullon 2002), caudate volume may be negatively affected by decreased DA release in later life. However, it should be noted that the relationship between DA receptor density and DA synthesis/release still remains unclear. A recent PET study showed a positive relationship between DA synthesis capacity and striatal D2/3 binding (Berry et al. 2017), while Ito et al. (2011) found this association to be negative. Animal studies indicate that DA D2 receptors can modulate innate immunity through αB-crystallin, which can reduce neuroinflammation (Shao et al. 2013). This suggests that lower D2 receptor density in A allele carriers may be associated with higher levels of neuroinflammation, which is likely to alter caudate volume. The link between striatal DA receptor density and brain morphology is further supported by studies showing an association between D2 receptor density and grey matter volume. Woodward et al. (2009) investigated the association between D2 receptor distribution and morphology, using positron emission tomography and structural MRI. They demonstrated a positive relationship between D2 binding potential and grey matter volume in bilateral caudate nucleus. Thus, larger caudate volume in ANKK1 Taq1A non-carriers, who presumably have higher striatal receptor density, might stem from the fact that denser and larger grey matter volume can support a higher number of D2 receptors. Alternatively, D2 receptor density and, by extension, DA availability, may alter structural brain integrity.

To our knowledge, this is the first study to investigate the effects of the ANKK1 Taq1A polymorphism on caudate volume in a large sample of older adults. Previous studies in younger adults have shown inconsistent results regarding the impact of this polymorphism on brain morphology. For example, in younger adults, Cerasa et al. (2009) found decreased midbrain volume in A-carriers compared to non-carriers, but no significant group difference was found in the striatum. The reason for this lack of group difference could be insufficient power due to relatively small sample sizes, and the fact that only younger participants were examined. However, another study with larger samples did not find any A-allelic effect on striatal volumes in older people (Stein et al. 2011). In that particular study, however, the whole sample (734 persons) also included participants with MCI (357 subjects) and AD (172 subjects). A possible explanation for the findings from Stein et al. (2011), and in line with the resource-modulation hypothesis, is that genetic effects diminish once individuals reach very low brain-resource levels, such as in MCI or AD. Therefore, potential effects of the A allele on caudate volume might be diluted by including both older adults without dementia and memory-impaired individuals in the same sample. An additional reason for a lack of genetic effects on caudate volume in previous studies could be that our results show that the genetic effect is only significant in people older than 66 years. Thus, including younger elderly in these studies may also attenuate the relationship between the ANKK1 Taq1A polymorphism and brain structure.

Caudate DA neuromodulation is critical for working memory (Erixon-Lindroth et al. 2005), perceptual speed (Bäckman et al. 2000), and episodic memory (Nyberg et al. 2016). However,we found no effect of the ANKK1 gene on any of the cognitive measures investigated. Previous studies are inconsistent regarding the genetic effect on cognition. Past work has reported that the A allele of the Taq1A polymorphism was associated with worse cognitive performance (Berryhill et al. 2013; Jocham et al. 2009; Persson et al. 2015), whereas others have failed to document such an effect (Bartres-Faz et al. 2002; Cerasa et al. 2009). There are several possible reasons for the inconsistent findings. First, cognitive functioning is influenced by complex interactions among a variety of brain regions and shaped by a multitude of genetic polymorphisms. Our study examined the effects of a single genetic polymorphism, which may contribute only a small proportion of the variance in cognitive performance. Second, measures of brain structure and function may be more sensitive to genetic effects compared to behavioral measures, as brain parameters are more proximal to the underlying molecular mechanisms and therefore more directly affected by a genetic polymorphism (Rasch et al. 2010; Harris and Deary 2011; Mattay et al. 2008; Hariri et al. 2006).

Some limitations of the current study should be noted. First, although we found a reliable influence of the DRD2/ANKK1 polymorphism on caudate volume in this relatively large sample of older adults, it is not possible to provide direct evidence to the view that the A allele of this particular polymorphism causes smaller caudate volume, because of reduced DA release. Second, we performed analyses on the left and right caudate volumes separately. Given that this additional analysis includes many independent tests, it renders multiple comparison correction necessary. To overcome this problem, we performed the repeated-measures ANCOVA on caudate volume for laterality × age × gene. Results from this analysis showed a significant laterality × age × gene interaction and a significant age × gene interaction only for the right caudate. This approach controls for the multiple chances to find differences, and it does so without assuming independence of the different variables. Furthermore, our results are based on cross-sectional data, so we were not able to estimate the rate of longitudinal brain changes and also cannot separate age effect from cohort and period effects. Large-scale longitudinal studies of healthy elderly persons are needed to substantiate the present findings.
